# Vessel‐on‐a‐Chip to Study Vascular Endothelial Inflammation

**DOI:** 10.1002/cpz1.70281

**Published:** 2025-12-18

**Authors:** Svitlana M. Palii, Anastasiia Voytovych, Nadiya Muzyka, Nuria Chantada, Pablo J. Sáez, Ezequiel Álvarez, Oksana Shevchuk

**Affiliations:** ^1^ Department of Pharmacology and Clinical Pharmacology I. Horbachevsky Ternopil National Medical University Ternopil Ukraine; ^2^ Cell Communication and Migration Laboratory, Institute of Biochemistry and Molecular Cell Biology, Center for Experimental Medicine University Medical Center Hamburg‐Eppendorf Hamburg Germany; ^3^ Department of Medical Biochemistry I. Horbachevsky Ternopil National Medical University Ternopil Ukraine; ^4^ Department of Biology University of Wisconsin‐Madison Madison Wisconsin; ^5^ Departamento de Farmacología, Farmacia y Tecnología Farmacéutica Universidad de Santiago de Compostela Santiago de Compostela Spain; ^6^ Instituto de Investigación Sanitaria de Santiago de Compostela (IDIS) Complexo Hospitalario Universitario de Santiago de Compostela (CHUS), SERGAS, Travesía da Choupana s/n Santiago de Compostela Spain; ^7^ CIBERCV Instituto de Salud Carlos III Madrid Spain; ^8^ These authors contributed equally to this work

**Keywords:** E‐selectin, Human umbilical vein endothelial cells, tumor necrosis factor, Vessel‐on‐a‐chip

## Abstract

The complex network of blood vessels plays a key role in transporting oxygen and nutrients and maintaining homeostasis in the human body. The inner walls of all blood and lymphatic vessels are lined by the endothelium, a monolayer of endothelial cells (ECs) oriented along the direction of blood flow. ECs play a pivotal role in vascular homeostasis, including regulating vascular tone, delivering oxygen and nutrients, modulating pro‐inflammatory molecules and pro‐inflammatory immune responses, and performing other vital functions. Therefore, the study of EC biology and vascular responses is key for a deeper understanding of vascular biology and the development of new therapeutics. Most studies *in vivo* and *in vitro* present technical challenges, either complexity or oversimplification, respectively, which slow down advances in the field. Therefore, 3D models and microfluidics offer a complementary alternative that integrates shapes similar to those observed *in vivo*, with the advantages of an *in vitro* system. Here, we present a robust and reproducible vessel‐on‐a‐chip (VOC) composed of an EC monolayer and a microvascular microenvironment maintained by a peristaltic pump to ensure continuous media circulation and physiological levels of shear stress. In addition, we validated this model for *in vitro* studies of vascular inflammation by monitoring EC status. We observed cellular alignment after shear stress exposure, increased E‐selectin expression, and TNF‐induced morphological changes in ECs. This new VOC is a promising approach to studying EC mechanobiology and inflammation and opens new avenues for its versatile use in vascular biology, inflammation, and immune and cancer cell migration in a controlled, scalable manner. © 2025 The Author(s). Current Protocols published by Wiley Periodicals LLC.

**Basic Protocol 1**: 3D Vessel Formation within a microfluidic organ‐on‐a‐chip system

**Basic Protocol 2**: Evaluation of shear stress

**Basic Protocol 3**: Evaluation of inflammation

## Introduction

In the human body, an intricate network of vessels is vital for maintaining homeostasis (Egawa et al., 2013). The endothelium lines the lumen of vessels; it consists of endothelial cells (ECs) and establishes a semipermeable barrier between the luminal contents and the tissue interstitium (Egawa et al., 2013). Thus, ECs play a vital role in regulating blood flow and vascular tone, maintaining blood fluidity, delivering oxygen and nutrients, modulating the sensing and release of pro‐inflammatory molecules, and consequently regulating the immune response and hemeostasis by preventing thrombosis through various anticoagulant and antiplatelet mechanisms (Jia et al., 2019; Medina‐Leyte et al., 2021; Trimm & Red‐Horse, 2023). Thus, a deeper understanding of the mechanisms regulating EC biology and associated vascular responses is necessary to lay the foundation for targeted and effective therapeutic strategies.

The endothelium plays a critical role during inflammation or infection, as it serves as the boundary between the blood and surrounding tissues. The endothelium is activated by inflammatory mediators such as tumor necrosis factor (TNF), previously known as TNF‐α (Grimstad, 2016), which triggers the expression of cell adhesion molecules (CAM), including selectins in ECs (Lee et al., 2013). To study the role of the endothelium, a broadly used *in vitro* method is based on the isolation of Human umbilical vein endothelial cells (HUVECs), because they are a reliable source of ECs with a proven capacity for capillary morphogenesis as shown in *in vitro* models (Bezenah et al., 2018), they retain their migratory potential (Ron et al., 2024), and expression of key molecules that are required for EC function (Villalobos‐Labra et al., 2018). They demonstrate consistent results regardless of the isolation method and have angiogenic properties similar to those of other EC types, such as Human microvascular endothelial cells (HMVECs) (Bezenah et al., 2018).

*In vivo*, blood vessels are studied using various advanced imaging techniques (Egawa et al., 2013; Kisler et al., 2018; Silver, 2021; Phinikaridou et al., 2016; Radu & Chernoff, 2013). Nevertheless, despite the progress of these techniques, they still present limitations (e.g., in drug screening), which can be overcome by using in vitro models that provide controlled, more reproducible microenvironments. Similarly, despite their advantages, these *in vitro* models also have limitations as we discuss next. The most common model for drug screening is the 2D cell culture. However, because cells are grown on a flat surface, this model fails to mimic key properties of vessels and often shows responses that differ from those of 3D models (Jensen & Teng, 2020). They typically do not incorporate dynamic features such as fluid flow, and cannot often model multicellular interactions, three‐dimensional architecture, and the mechanical forces characteristic of native tissues. In contrast, organoids and 3D cell cultures are considered to mimic several key features of blood vessels and other tissues (Leung et al., 2022).

In particular, organ‐on‐a‐chip (OoC) approaches allow more accurate replication of human physiopathology, as they enable real‐time monitoring of multiple parameters while testing the effects of various types of perturbations, i.e., genetic or pharmacological (Jensen & Teng, 2020). Several vascular OoC models are based on the self‐assembly of networks (Kim et al., 2013, 2016; Lee et al., 2024), or use microfluidics as a scaffold (Ansarizadeh et al., 2025; Dessalles et al., 2022; Lugo‐Cintrón et al., 2020; Marder et al., 2024; Menon et al., 2017; Riddle et al., 2024; Zhang et al., 2016). Some models allow fluid circulation to study EC mechanobiology by imposing fluid shear stress, cyclic stretching, and hydrostatic pressure (Hu et al., 2019). Altogether, these models allow the study of angiogenesis in both physiological and pathological conditions (Costa et al., 2020; Simitian et al., 2022; Shakeri et al., 2023), which is a significant advantage for the study of rare genetic diseases. Unfortunately, most of these models are not well‐suited for broad use by non‐specialists, which creates challenges for reproducibility. OoC technologies will likely become the standard tool for biomarker discovery and drug screening in the near future, allowing faster development of personalized medicine (Srivastava et al., 2024). Therefore, it is necessary to increase the technical feasibility of using these models.

Here, we present a reproducible system for producing 3D vessels with an incorporated circulation system that enables the study of wall shear stress (WSS) and the injection of chemical compounds. We validated this vessel‐on‐a‐chip (VOC) model by evaluating the effects of WSS on endothelial integrity and the effects of inflammation on ECs under flow. We effectively demonstrate the vascular integrity and EC alignment induced by shear stress and TNF‐induced vessel inflammation, as evidenced by E‐selectin expression, a CAM, and morphological changes in ECs.

## Cautions

The entire circuit should be cleaned and sterilized before the experiment.

The flow circuit should be assembled in a laminar flow hood under sterile conditions.

*NOTE*: All protocols involving animals must be reviewed and approved by the appropriate Animal Care and Use Committee and must follow regulations for the care and use of laboratory animals. Appropriate informed consent is necessary for obtaining and use of human study material.

## Basic Protocol 1 3D VESSEL FORMATION WITHIN A MICROFLUIDIC Organ‐on‐a‐Chip SYSTEM

This protocol enables the work with a dynamic VOC model based on a monolayer of HUVEC cells prepared for drug and/or inflammatory factor testing under flow conditions. This system closely mimics the human vasculature by creating a vessel‐like structure connected to a peristaltic pump via silicone tubing, ensuring continuous circulation.

### Materials

Fibronectin (Sigma–Aldrich, cat. no. 11051407001)
Gelatin Type B (Sigma–Aldrich, cat. no. 097K0108)
Endothelial growth medium‐2 (EGM‐2) (Lonza, cat. no. CC‐4176)
Human umbilical vein endothelial cells (HUVECs): obtained after informed consent of the donors under the frame of a research project authorized by the local Human Research Ethics Committee in Galicia, in full accordance with the Declaration of Helsinki of the World Medical Association. Alternatively, commercial human endothelial cells can be used.
0.25% Trypsin‐EDTA (Thermo Fisher, cat. no. 25200‐056)

Laminar flow hood
Bifurcated Vessel‐on‐a‐Chip (BFlow)
Silicone tubing 1 mm ID, 3 mm OD (BFlow, cat. no. B02_0009)
Y Tube Fitting connector, 1.6 mm ID (Ibidi, cat. no. 10828)
3‐stop platinum‐cured silicone tubing 1.65 mm ID, 3 mm OD (Darwin Microfluidics, cat. no. SE‐TUB‐SIL‐SSS‐1*1)
Luer Lock Connector Male (Ibidi, cat. no. 10826)
Female Luer Lock Coupler (Ibidi, cat. no. 10823)
Straight Chip Connector (BFlow)
Glass jar
20G Syringe needle (BFlow, cat. no. B02_0024)
Spinal needle (long needle) (75 mm; 18G; R. Costoya, cat. no. BDAM405247)
Reservoirs with septum cap (ThermoScientific, cat. no. 047422‐4001)
Cell culture maintenance incubator (37°C, 5% CO_2_, humidified; Phcbi)
Straight Press‐in Plug (BFlow, cat. no. B02_0029)
Reglo independent channel control (ICC) peristaltic pump (Ismatec, cat. no. MFLX78018‐24‐EU)
Cell culture test incubator (37°C, 5% CO_2_, humidified; New Brunswick, Galaxy 170 S)

#### 3D Vessel‐on‐a‐chip

**Figure 1:**
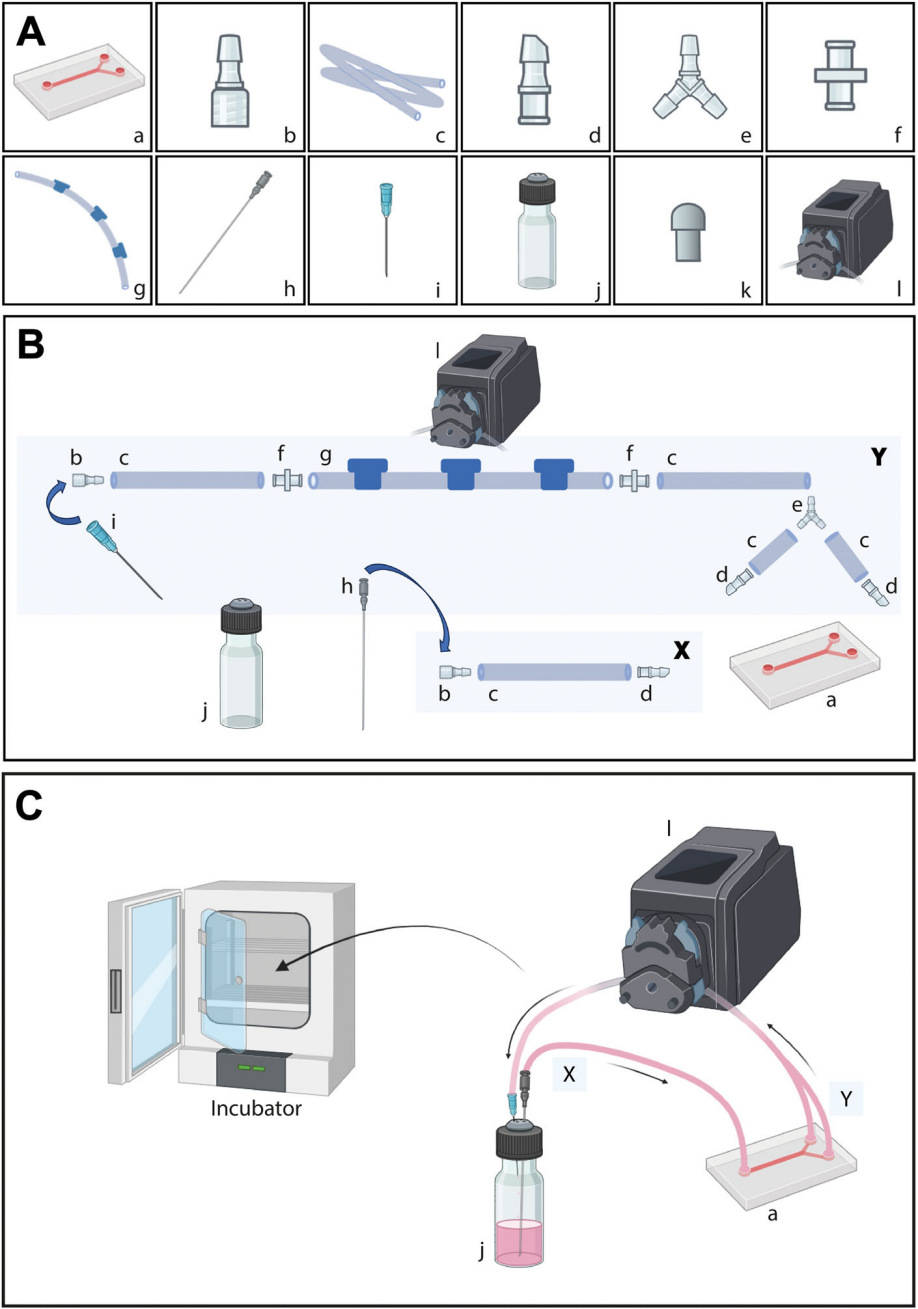
Schematic representation of the experimental setup underflow. (**A**): Individual elements used in the assembly of a circuit flow: (**A**) a ‐ Bifurcated Vessel‐on‐a‐Chip; b ‐ Luer Lock Connector Male; c ‐ Silicone Tubing; d ‐ Straight Chip Connector; e ‐ Y Tube Fitting; f ‐ Female Luer Lock Coupler; g ‐ 3‐stop Platinum‐cured Silicone Tubing; h ‐ spinal needle (long needle); i ‐ 20G Syringe needle; j ‐ Glass Vial with Rubber Stopper; k ‐ Straight Press‐in Plug; l ‐ Peristaltic Pump. (**B**) Schematic depiction of the tube assembly, consisting of two sections (X and Y). X represents the tube connecting the reservoir to the inlet of the VOC, composed of materials “b+c+d”, as shown in **Figure 1**A. Y corresponds to the tube running from the chip through the pump and back to the reservoir, consisting of materials “d+c+e+c+b+f+b+g+b+f+b+c+b”, as indicated in **Figure 1**A. (**C**) A schematic representation of the assembled system and its functionality, with arrows indicating the direction of flow. The reservoir with the medium is also shown, functioning to prevent bubble circulation within the system.

1 Prior to sterilization, gently rinse the Bifurcated VOC (see Fig. 1A ‐ a) twice using sterile distilled water.

2 After washing, join the silicone tubes and connectors (see Fig. 1B) to form two separate parts, labelled “X” and “Y.” Part “X” represents the tube connecting the reservoir to the inlet of the VOC, and includes the Luer Lock Connector Male (see Fig. 1A ‐ b), Silicone Tubing (Fig. 1A ‐ c), and Straight Chip Connector (Fig. 1A ‐ d), as shown in Figure 1B. Part “Y” corresponds to the tube running from the chip through the pump and back to the reservoir, via the Straight Chip Connector (Fig. 1A ‐ d), Silicone Tubing (Fig. 1A ‐ c), Y Tube Fitting (Fig. 1A ‐ e), Silicone Tubing (Fig. 1A ‐ c), Luer Lock Connector Male (see Fig. 1A ‐ b), Female Luer Lock Coupler (Fig. 1A ‐ f), Luer Lock Connector Male (see Fig. 1A ‐ b), 3‐stop Platinum‐cured Silicone Tubing (Fig. 1A ‐ g), Luer Lock Connector Male (see Fig. 1A ‐ b), Female Luer Lock Coupler (Fig. 1A ‐ f), Luer Lock Connector Male (see Fig. 1A ‐ b), Silicone Tubing (Fig. 1A ‐ c), and Luer Lock Connector Male (see Fig. 1A ‐ b), as indicated in Figure 1B.
3 Autoclave the components as follows: place the PDMS microchannel in a glass jar and autoclave at 121°C before the experiment. Autoclave the following elements in separate glass jars: inlet (see Fig. 1A ‐ h) and outlet (see Fig. 1A ‐ i) needles, the glass reservoirs fitted with rubber septa in their caps (see Fig. 1A ‐ j), and parts “X” and “Y”, which contain silicone tubes and connectors.
4 To help support cell attachment, the channels are coated with fibronectin (FN) one day before cell seeding. Channels are treated with 5 µg/ml FN prepared in 0.02% gelatin, Type B, avoiding bubble formation (see Table 1). The volume capacity per channel of the Bifurcated VOC is 110–120 µl. Incubate the device overnight in a humidity‐saturated atmosphere at 37°C with 5% CO_2_.
  It is critical to prevent bubble formation at this step. Any remaining bubbles will leave areas of the channel unfilled with the coating solution, and the cells will not adhere in subsequent steps. To avoid bubble formation during incubation, a “straight press‐in plug” (see Fig. 1A – k) is recommended.

**Table 1:** Troubleshooting Guide for Connecting the VOC to the Circulation System

Problem	Possible cause	Solution
Bubbles when filling the chip with FN	1. Discontinuous injection of FN 2. Incorrect tip size	1. Introduce FN gradually and without stopping, slowly 2. Change tip size
Connected the system, but the chip is not filled with medium	Straight Chip Connector not inserted correctly into the Сhip	Make sure the cut on the connector faces the microvessel, not the opposite side, which prevents medium from passing through.
One arm (outlet silicone tube) is not filled with medium	The outlet tubes are not placed at the same height	Clamp the opposite outlet silicone tube until the empty arm is filled and flowing.
Medium does not enter the tube system from the glass reservoir	Clogged needle	Change to a new needle

5 Once the channel has been coated and incubated, it is ready for seeding with HUVECs. Gently aspirate FN from the microfluidic channel. If necessary, the channel may be rinsed once with EGM‐2.
6 Trypsinize and carefully detach cells from an 80%–90% confluent culture dish.
7 Count the collected cells and evenly seed 1 × 10^6^ cells/ml in EGM‐2 onto the upper side of the channel, ensuring uniform distribution across the surface. Cells should be left to adhere for 4–5 hr.
8 Gently trypsinize and detach cells from a second 80%–90% confluent dish and seed onto the bottom surface of the channel using the same cell density and conditions.
9 Once the cells have attached, carefully replace the medium in the chip with fresh medium.
10 When conducting the experiment within an incubator, where observation is not possible, evaluate cell viability under a microscope prior to initiating flow. Document the condition of the cells to establish a baseline for post‐flow comparison.
11 Once the steps listed above are completed, assemble the flow circuit (see Fig. 1B). Connect the “X” part to the inlet of the microchannel and the “Y” part to the outlet, ensuring that “d” (see Fig. 1A) properly links the chip with the silicon tubing. Next, join the inlet (see Fig. 1A ‐ h) needle to the Luer Lock Connector Male of part “X” and then to the glass reservoir. The outlet (see Fig. 1A ‐ i) needle is connected with the Luer Lock Connector Male of part “Y”, but not to the reservoir at this point.
12 Assemble a 3‐stop Platinum‐cured silicone tube in a peristaltic pump (see Fig. 1A ‐ l). Start filling the system with medium at a minimum speed of 0.116 ml/min. When the silicone tube circuit and chip are filled with medium, stop the peristaltic pump and connect the short needle to the glass reservoir (see Fig. 1C). The reservoir is positioned just before the chip, serving as a bubble trap to prevent air from entering the channel.
13 Transfer the setup to the incubator or microscope stage, maintaining a humidified 5% CO_2_ and 37°C environment for cell culture. Take care to avoid tipping the reservoir, as this may introduce air bubbles into the circulation and adversely affect the cultured cells. Also, avoid disconnecting the silicone tubes from the chip, as this may cause medium leakage and introduce air bubbles into the system.
14 Once everything is properly connected and stabilized, turn on the peristaltic pump at a low flow rate of 0.116 ml/min. After the cells have had time to adapt, increase the flow to a maximum of 0.75 ml/min. This flow rate should be maintained throughout the experiment.
  During the experiment, treatments (such as drugs for testing) or circulating cells can be added as required, while maintaining the desired flow conditions for at least a few hours. An insulin syringe ought to be used to introduce the treatment into the system, using the septum in the glass reservoir as indicated above.
  If needed, cells should be fixed immediately after stopping the flow. Fixation should follow the specific protocol required for downstream staining or analysis.
  If supernatant collection is required, it should be recovered promptly and stored under appropriate conditions, as specified by the experimental requirements.

## Basic Protocol 2 EVALUATION OF SHEAR STRESS

Shear stress is a tangential force acting on the EC surface, caused by friction between the vessel wall and the circulating blood, in our case, the EGM‐2. Shear stress for arterial ECs is 10–40 dyne/cm^2^ and 1–6 dyne/cm^2^ for venous ECs (Zeng et al., 2018). To assess the impact of shear stress on EC integrity within the VOC system, two physiologically relevant WSS conditions were applied: a low shear stress of 0.2 dyne/cm² and a higher shear stress of 1.3 dyne/cm², corresponding to a typical venous flow rate. Both flow conditions were maintained continuously for 24 hr.

**Figure 2:**
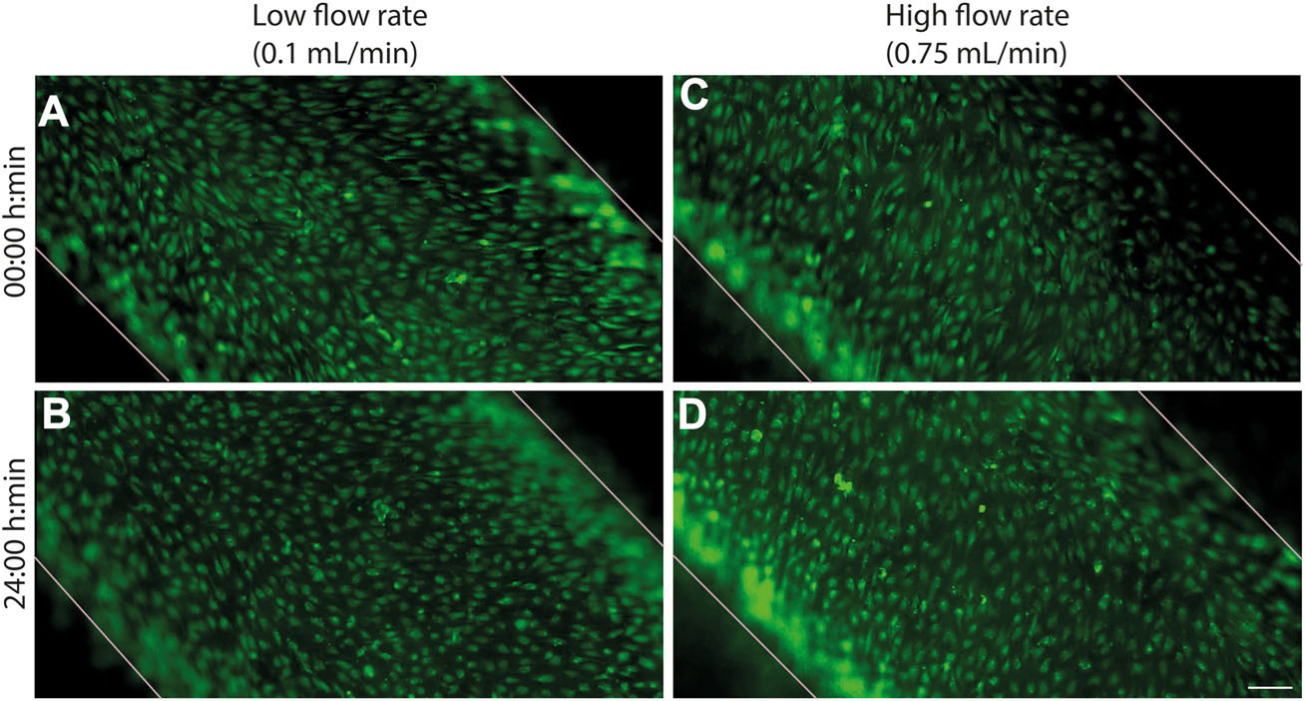
Flow rate modulates endothelial integrity in a VOC model. Endothelium stabilization and alignment induced by low and high flow. (**A and C**) EC morphology before the flow. (**B**) Endothelium after 24 hr of low flow (0.116 ml/min), showing a stable monolayer. (**D**) Endothelium after 4 hr of low flow followed by 20 hr of high flow (0.75 ml/min), showing an intact monolayer and early signs of flow‐induced alignment. Scale bar: 100 µm.

Throughout the experimental period, EC morphology and confluence were monitored using fluorescence microscopy. At both shear stress conditions, the endothelial monolayer remained intact, confluent, and uniformly distributed along the microchannel surface. No visible gaps, detachment, or signs of endothelial damage were detected. In the low WSS condition (0.2 dyne/cm²), ECs showed a typical cobblestone‐like morphology. Under higher shear (1.3 dyne/cm²), cells began to exhibit mild elongation and alignment in the direction of flow, consistent with known flow‐induced cellular polarization. These findings confirm that both shear stress values were well tolerated by the ECs, preserving monolayer integrity and viability within the time frame tested. This demonstrates the suitability of the applied shear stress protocol for subsequent experiments involving inflammatory stimulation or drug treatment under flow conditions. Microscopy images illustrating EC monolayer conditions before and after shear effect are presented in Figure 2, panels A–D.

### Additional materials (also see Basic Protocol 1)

Calcein, AM, a cell‐permeant dye (ThermoFisher, cat. no. C1430)

Hoechst 33342 (NucBlue^TM^ Molecular Probes, Thermo Fisher, cat. no. R37605)
Olympus IX 73 P2F inverted microscope

1 Prepare at least two VOCs to compare the influences of shear stress on ECs.
2 Complete steps 1–5 of Basic Protocol 1 to establish a 3D vessel.
3 Stain the cells with Calcein AM, a cell‐permeant viability dye [1:1000] in EGM‐2 for 20 min at 37°C, 5% CO_2_, and Hoechst 33342 [1:1000] in EGM‐2 for 20 min at 37°C, 5% CO_2_.
4 Check cell viability under a microscope to establish a baseline prior to initiating flow.
5 Follow steps 7–9 of Basic Protocol 1 to establish a proper connection between the VOC and the peristaltic pump.
6 Once everything is properly connected and stabilized, turn on the peristaltic pump. Choose the flow rate for testing.
7 Start the peristaltic pump and maintain the set flow rate for at least 24 hr to assess the effect of shear stress on the endothelium.
  If needed, fix the cells immediately after stopping the flow. Fixation should follow the specific protocol required for downstream staining or analysis.
  If supernatant collection is required, it should be recovered promptly and stored under appropriate conditions, as specified by the experimental requirements.

## Basic Protocol 3 EVALUATION OF INFLAMMATION

ECs maintain constant, direct contact with the bloodstream and serve as primary targets for viral and bacterial infections. TNF is considered a master regulator of inflammatory reactions, plays an important role in endothelial dysfunction, and is also an important player in the pathogenesis of some autoimmune and chronic inflammatory diseases, since inappropriate or excessive activation of TNF associated with chronic inflammation can lead to the development of pathological complications (Horiuchi et al., 2010; Jang et al., 2021). TNF is a potent proinflammatory cytokine that has pleiotropic effects on various cell types. TNF activates ECs to express and release various inflammatory chemokines, cytokines, and adhesion molecules (Medina‐Leyte et al., 2021). E‐selectin, also known as CD62E, is a CAM expressed on cytokine‐activated ECs (Huang et al., 2021). Under normal conditions, E‐selectin, P‐selectin, and VCAM are not expressed on the surface of ECs; rather, they are found on activated endothelium, for example, in response to inflammatory cytokines such as TNF (Moore et al., 2021), IL‐1β, or lipopolysaccharide (LPS) (Huang et al., 2021; Ogrodzinski et al., 2023). However, some studies have found its expression in unstimulated ECs in vitro (Jubeli et al., 2012).

**Figure 3:**
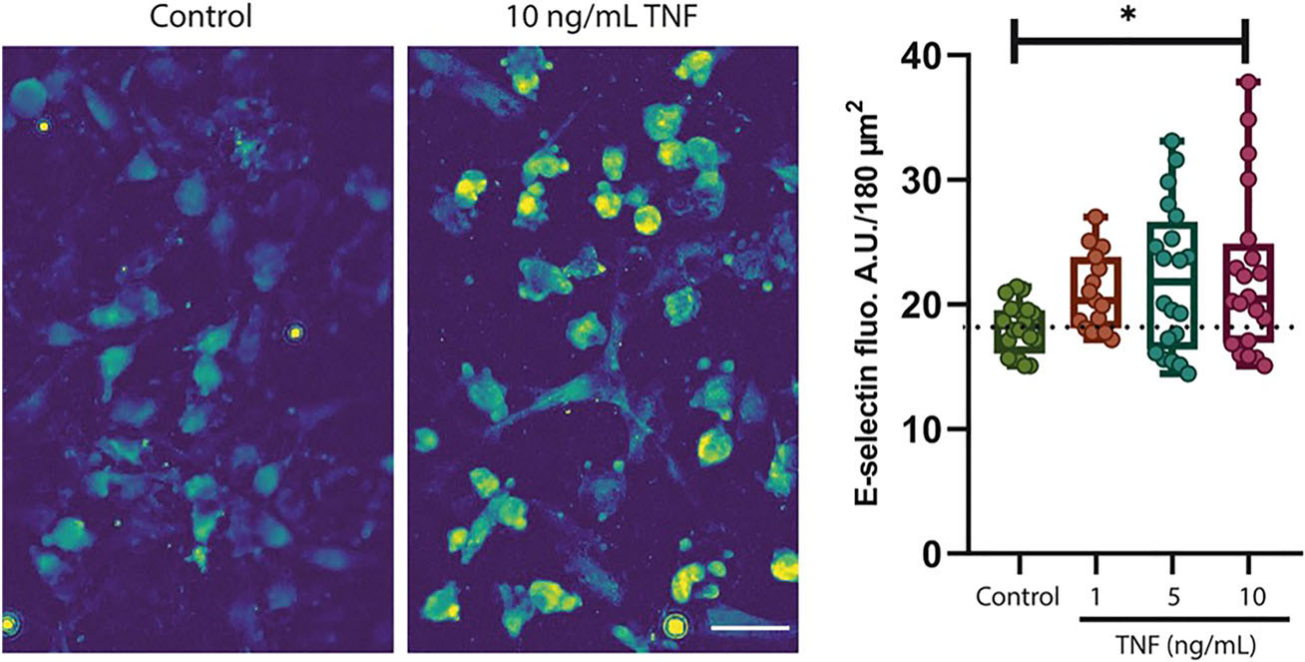
TNF induces E‐selectin expression in endothelial cells. *Left*, E‐selectin immunoreactivity in ECs in the VOC model. Note the increased abundance of E‐selectin after 18 hr treatment with 10 ng/ml TNF, which indicated endothelial activation. Scale bar: 100 µm. *Right*, quantification of E‐selectin immunoreactivity. The box plot shows the median, the bars show the min/max range, and the dotted line depicts the median of the control. Ordinary one‐way ANOVA, Tukey's multiple comparisons test. *P < 0.05 compared to control condition, n > 20 cells per condition of a representative experiment.

The evaluation of inflammation was carried out according to this protocol using different TNF concentrations. Four groups were established: (1) Control, (2) 1 ng/ml TNF, (3) 5 ng/ml TNF, and (4) 10 ng/ml TNF. Our study found that the fluorescent signal for E‐selectin was significantly higher in the 10 ng/ml TNF group than in the control, *p* = 0.04. In groups with 1 and 5 ng/ml TNF, there was no significant increase in intensity, with *p* values of 0.33 and 0.06, respectively (Fig. 3).

### Additional materials (also see Basic Protocols 1 and 2)

Recombinant Human TNF (Gibco, cat. no. PHC3016)
PBS (Merck, P3813)
4% (w/v) paraformaldehyde (AlfaAesar, cat. no. J67899)
0.2% (v/v) Triton X‐100 (Sigma, cat. no. X100)
4% bovine serum albumin (BSA) (Sigma, cat. no. A7906)
Anti‐ESELE (R&D Systems, cat. no. BBA16)
Alexa Fluor 568 fluorescence‐conjugated anti‐rabbit IgG secondary antibody (ThermoFisher, cat. no. A‐11011)

Insulin syringe U‐100 with needle.

**Figure 4:**
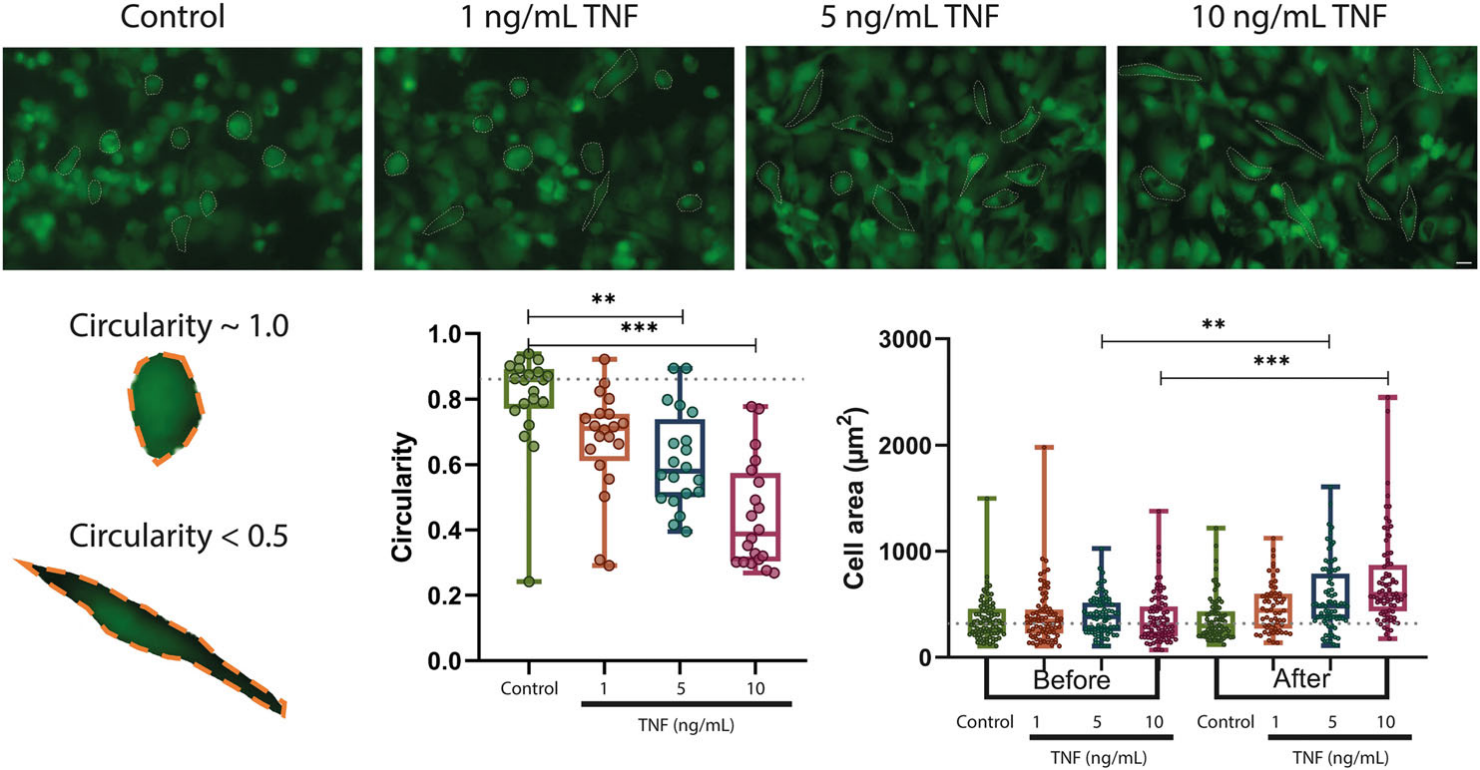
TNF increases EC area and alters cell morphology. *Top*, microscopic images of ECs in VOC stained with calcein after treatment for 18 hr with TNF at indicated concentrations (1, 5, 10 ng/ml). Scale bar: 100 µm. *Bottom*, scheme to depict the analysis of circularity. Quantification of the data shown in the images upon TNF stimulation. Note that TNF induced a reduction in circularity, showing cell elongation, and an increase in surface area as cell density decreased, suggesting hypertrophic changes or morphological remodeling in response to inflammatory stress. The box plot shows the median, the bars show the min/max range, and the dotted line depicts the median of the control. Ordinary one‐way ANOVA, Tukey's multiple comparisons test. **P < 0.02, ***P < 0.001 between indicated treatments; n = 20 cells, N = 4 independent experiments, of which for circularity a representative experiment is shown.

1 Prepare four VOCs to evaluate the inflammation of the different concentrations of inflammatory mediators, for example, TNF on ECs.
2 Steps 1–5 of Basic Protocol 2 should be completed to establish a 3D vessel and to establish a proper connection between the VOC and the peristaltic pump.
3 Once everything is properly connected and stabilized, turn on the peristaltic pump at a low flow rate of 0.116 ml/min. After the cells have had time to adapt for at least 4 hr, increase the flow to maximum at 0.75 ml/min. This flow rate should be maintained throughout the experiment.
4 A variety of pro‐inflammatory soluble molecules can be used to mimic inflammation. To introduce a treatment into the system, use a U‐100 insulin syringe for precise dosing.
  It is recommended to administer TNF through the septum of the glass reservoir (see Fig. 1A ‐ j) to ensure sterile, direct entry into the circulation.
  To maintain pressure balance within the circuit, a separate syringe needle (see Fig. 1A ‐ i) without an attached syringe should be temporarily inserted through the septum to allow pressure equilibration. This equilibration needle should be removed immediately after the treatment has been added to prevent contamination or pressure disturbances. The experiments used to determine the final TNF concentration are described below.
  Concentration should be selected for each cell type.
5 After stopping the flow, wash the cell‐seeded channel with phosphate‐buffered saline (PBS).
  If supernatant collection is required, it should be recovered promptly and stored under appropriate conditions, as determined by the experimental requirements.
6 Fix the cells immediately with 4% (w/v) paraformaldehyde in PBS for 15 min at RT.
7 After fixation, permeabilize the cells with 0.2% (v/v) Triton X‐100 in PBS for 15 min at RT.
8 After blocking with 4% bovine serum albumin (BSA, Sigma) in PBS for 1 hr at RT, incubate the VOC with primary antibodies for E‐selectin [1:200], 1 hr at RT, followed by secondary antibodies for Alexa Fluor 633 [1:500], 2 hr at RT.
9 Wash the VOC three times with PBS‐T (PBS with 0.05% Tween 20).
  If needed, the VOC can be stored at 4°C before imaging.
10 Transfer the VOC to the microscope stage for imaging.
11 Analyze the acquired images using Fiji.
12 To quantify E‐selectin (see Fig. 3), *select the rectangle tool > Draw ROI (˜180 µm^2^) > Analyze > Set measurement > Select mean grey value > On the keyboard, press M*. Select five squares in various image areas measuring intensity in these ROI. Transfer data to Excel and perform statistical analysis using statistical software.
13 To count the number of cells, *select the rectangle tool > Draw ROI (˜380 µm^2^) > On the keyboard, press Ctrl + Shift + D > Select multi‐point > Calculate the number of nuclei in these ROI*. Select four more random squares in different areas of the image and repeat the process.
14 To analyze the cell area (see Fig. 4), *measure the parameters by activating in Fiji > Analyze > Set measurement > Select what needs to be measured (for example, area, shape descriptors, centroid, etc.) > select the freehand line tool > draw ROI around the cell > on the keyboard, press the M key*. Detailed analyses of shape descriptors have been previously reported (Sáez et al., 2018). Transfer the data to an Excel file (or a similar program) to create the graphs and perform the statistical analysis. It is recommended to use statistical software to plot the data and obtain a refined statistical analysis. Analyze the cell area in at least 20 random cells from different areas of each image. Since an ROI was drawn around the cell in this step, several morphological parameters can be evaluated.

## Reagents and Solutions

### AlexaFluor 568‐conjugated secondary antibody

Dilute 1:500 in PBS‐T. Prepare fresh and use immediately.

### Bovine Serum Albumin, 4%

Prepare 4% bovine serum albumin (BSA) in PBS. Store no more than 2 weeks at 4°C to prevent bacterial growth. Keep protected from light.

### Calcein, AM

Dilute Calcein, AM, 1:1000 in EGM‐2. Use immediately.

### E‐selectin primary antibody

Dilute 1:200 in PBS‐T. Prepare fresh and use immediately.

### Fibronectin, 5 µg/ml

Prepare 5 µg/ml Fibronectin in 0.02% gelatin, Type B. Use immediately.

### Hoechst 33342

Dilute Hoechst 33342 1:1000 in EGM‐2. Use immediately.

### Human TNF

Prepare dilutions of recombinant Human TNF at 10 ng/ml, 5 ng/ml, and 1 ng/ml in EGM‐2 and use immediately.

### Paraformaldehyde, 4%

Prepare 4% (w/v) paraformaldehyde in PBS. Store up to 3 months at 4°C, protected from light.

### PBS‐T

Add 0.5 ml Tween 20 to 1 L of 1× PBS and mix well. Adjust to pH 7.4 with HCl and NaOH. Store at room temperature for up to 1 week.

### Triton X‐100

Prepare 0.2% (v/v) Triton X‐100 in PBS. Seal tightly and store in a cool, dark place to prevent oxidation.

## Commentary

### Background Information

Depending on the research question, scientists use different models to study ECs. One‐dimensional (1D) models (Ron et al., 2024) are used to analyze the behavior of individual cells, observing morphology, cytoskeletal organization, studying signaling pathways activated during migration, and assessing the nature of migration itself (J. Liu et al., 2024). The scratch assay (also known as the wound healing assay) is a classic model for studying collective cell migration in two‐dimensional (2D) cultures (Gov, 2007; Li et al., 2013; Vitorino & Meyer, 2008). This technique is used to study the coordinated movement of a cell population and is considered the *in vitro* standard for analyzing collective cell migration in a 2D environment (Azzam et al., 2024; Jonkman et al., 2014; Stelling‐Férez et al., 2023; Suh et al., 2022). Three‐dimensional (3D) models enable the simultaneous consideration of vascular stiffness and the softness of surrounding tissues on cell behavior. Specifically, these conditions significantly affect cancer cell invasion, modulating their ability to penetrate and migrate through vascular and interstitial barriers (Uroz et al., 2024). In cardiovascular research, 3D spheroid cell cultures are an important experimental model that provides a more physiologically relevant environment than traditional 2D cultures, as they reproduce the spatial organization of cells and intercellular interactions observed *in vivo*. 3D spheroids serve as an effective model for analyzing angiogenesis, reproducing features of the cardiac microenvironment, and advancing the development of new pharmacological approaches and methods for cardiac tissue regeneration (Krug et al., 2025). Thus, current approaches to studying EC function, interactions with other cells, and migration range from overly simplistic 1D and 2D models (such as micropatterns or scratch assays) to highly complex *in vivo* systems. Instead, there is a need for models of intermediate complexity. One promising approach is the use of VOCs, which bridge this gap by offering greater physiological relevance than 1D and 2D systems while remaining less complex and more reproducible than *in vivo* models.

OoC is a technology that reproduces the structure of organs in microchips and has already successfully modelled gut (Ofori‐Kwafo et al., 2025), lungs (K. Li et al., 2019), liver (Yang et al., 2022), kidneys (Nguyen et al., 2023), heart (B. Liu et al., 2025), brain (Rodrigues et al., 2024), and skin (Zoio & Oliva, 2022). Combining multiple chips may allow the design of a “body‐on‐a‐chip” (Ying‐Jin et al., 2025). The use of flow in OoC devices allows the integration of the vascular system into simulated tissues, making it possible to analyze the spread of pathogens through the bloodstream, the migration (intravasation and extravasation) of immune and cancer cells, and the pharmacokinetics of drugs (Ehlers et al., 2023; Gaudreau & Stewart, 2024; Ma et al., 2018; Van Os et al., 2023; Zhang et al., 2022). In most OoC infection models, vascularization is achieved by the endothelial lining of a single compartment (Alonso‐Roman et al., 2024). Vascularized OoC models create conditions for reproducing controlled biological and physical influences that mimic the dynamic microphysiological environment of vessels *in vivo* (Lim et al., 2024). Emerging evidence shows that endothelial mechanobiology is a key factor in the progression of pathogenesis with vascular dysfunction. However, despite its relevance, due to a lack of appropriate technology and models, there remains a knowledge gap in understanding the relationship between physical stimuli, vascular stiffness, and shear stress during atherosclerotic plaque formation (Walther et al., 2021). In this context, VOC systems represent a promising platform for reproducing key mechanical parameters of the vascular microenvironment and investigating their contribution to the progression of pathological processes. For example, microfluidic shear‐tension devices provide various combinations of fluid shear stress and cyclic stretching (Chu et al., 2023) to simulate both physiological and pathological stretching in hypertensive vascular conditions (Y. Liu et al., 2025). Platforms with reproducible shear stress and flow conditions are used to study EC defects in slow‐flow venous malformations (VMs), assessing the response of EC orientation and area, actin organization, and Golgi polarization to unidirectional and bidirectional flow, and changes in WSS (Abe et al., 2019; Ansarizadeh et al., 2025). The creation of spatially complex 3D vascular models enables the reproduction of hemodynamic variations that directly affect the transport of blood and its components. Such models deepen understanding of intercellular and drug‐cell interactions under the influence of flow in both healthy and pathological conditions. Modelling vascular structures of different architectures allows for the reproduction of a wide range of anatomical features characteristic of human blood circulation. The designed vessels serve as platforms for studying complications associated with structural changes, in particular aneurysms, fibromuscular dysplasia, atherosclerosis, and others (Lee et al., 2025). Personalized OoCs with physiologically relevant parameters open the door to individualized drug evaluation and the development of treatment and prevention strategies. The main challenges for implementation are the access to personal samples, medical data, and treatment outcomes needed to validate the predictive value of such systems (van den Berg et al., 2019). Understanding how EC biology contributes to vascular responses will have a greater impact in the coming years as personalized OoC moves from academic research to practical applications in precision medicine. With the advent of advanced data collection methods, medicine is expected to become increasingly tailored to the individual, thereby shortening drug development timelines, lowering costs, and reducing adverse effects. OoCs can play a key role in this transition by providing a platform for experimental validation of personalized strategies (van den Berg et al., 2019). Therefore, the choice of model to study EC and its associated vascular responses will be key to advancing the field. In addition, to align with the principles of the 3Rs (Replacement, Reduction, and Refinement) for animal experimentation, there is an increasing need to use non‐animal or non‐*in vivo* models. In addition, the emergence of mechanobiology as a field of study has continued to reveal that 2D models do not always reproduce the *in vivo* situation (Bokhout et al., 2025; Hoarau‐Véchot et al., 2018; Wang et al., 2023). Therefore, new models are required to study EC and vessels in systems that reproduce as many characteristics as possible, including the cylindrical structure of vessels and the shear stress in levels close to those observed in pathophysiological conditions. Then, the combination of VOC with multiplexing imaging (Chen et al., 2025; Kuehl et al., 2025; Nguyen et al., 2024; Shevchuk et al., 2023; Xu et al., 2025; Zhao & Germain, 2023) could lead to the discovery of new pathways and regulators in health and disease.

In this protocol, we present a reproducible, scalable assay to monitor EC behavior within 3D vessels. Specifically, we describe the usage of this VPC to monitor the effects of (1) shear stress and (2) cytokine‐induced inflammation. We further describe the morphological changes observed in ECs during inflammation and reveal that TNF induces their elongation.

### Critical Parameters

The reliability of OoC experiments strongly depends on careful handling of both the device and the associated peristaltic flow system. Several critical parameters must be considered to maintain sterility, prevent technical failures, and preserve vascular integrity during long‐term experiments.

#### Labelling and identification

When multiple VOCs are used in parallel experiments, both the silicone tubing and the individual VOCs should be labelled clearly prior to autoclaving. This step minimizes the risk of confusion between experimental conditions (e.g., control vs. treatment groups) and ensures traceability throughout the workflow. Permanent, autoclavable labelling methods are preferred to avoid smearing or fading.

#### Device setup and inspection

Before initiating an experiment, all components of the system (as shown in Fig. 1) should be thoroughly checked to ensure proper placement and secure connections. Even minor misalignments or incomplete fittings can lead to leakage, air bubbles, or unstable flow dynamics. It is recommended to perform a pre‐experiment inspection and flow test at a minimum speed of 0.1 ml/min for 2 min with culture medium to confirm correct assembly.

#### Tubing management

The 3‐stop platinum‐cured silicone tubing should not be disconnected from the peristaltic pump once the system has been filled with medium. Disconnection may lead to fluid leakage, introduction of air bubbles, and contamination. If disconnection is unavoidable, additional clamps must be applied to the tubing prior to detachment to prevent backflow and air entry. After reconnection, the system must be carefully inspected, and any additional clamps should be removed to ensure a stable and uninterrupted flow.

#### Pump operation

Before activating the peristaltic pump, verify its direction and flow rate settings. Incorrect flow direction can expose ECs to non‐physiological stress or flush cells away from the channel. Flow rate settings should be validated in accordance with the experimental design to reproduce the intended WSS. Moreover, all tubing clamps must be released prior to pump activation, as residual occlusion may lead to overpressure, leakage, or localized flow disruption.

#### Medium handling

Adding or removing culture medium from the VOC must always be done slowly and carefully. Rapid aspiration or dispensing can disrupt the endothelial monolayer, increase shear stress beyond physiological levels, and cause partial cell detachment from the substrate. Using pipette tips positioned at an angle of 30° and applying minimal pressure reduces the risk of cell layer damage.

#### Air bubble prevention

The introduction of air bubbles into the microchannels is a frequent cause of cellular damage. Therefore, all tubing should be pre‐filled with medium before connection, and bubble traps (Fig. 1A, j) or filters are recommended.

#### Sterility considerations

Throughout handling, aseptic technique is mandatory. Both the tubing and VOC surfaces should remain sterile after autoclaving, and all manipulations should be performed under a biosafety cabinet. Repeated connections and disconnections increase the risk of contamination and should be minimized.

### Troubleshooting Table

Table 1

### Statistical Analysis

The data were analyzed using GraphPad Prism 8.0.1 with ordinary one‐way ANOVA and multiple comparisons (Tukey's test). The *P* values shown are as follows: *P < 0.05, **P < 0.02, and ***P < 0.001.

### Understanding Results

This model was intended not only to investigate EC responses to hemodynamic and inflammatory stimuli but also to lay the groundwork for subsequent pharmacological testing of therapeutic agents. Previous research in this field has primarily focused on clinical observations in patients (Bots et al., 2007) or on animal models. Although such approaches allow certain conclusions about endothelial function, they limit the ability to perform an in‐depth analysis of cellular mechanisms. Direct investigation of ECs *in vivo* is hindered by their anatomical location, a thin monolayer situated on the luminal surface of blood vessels, which is difficult to access for isolated analysis.

With advances in bioengineering, particularly in OoC technologies, it is now possible to create *in vitro* models with a high degree of physiological relevance. Notably, it has been demonstrated that EC surface receptor expression can differ significantly between conventional 2D cultures and more complex 3D dynamic systems that simulate *in vivo* conditions (Chiu et al., 2007; Ingber, 2022). In most studies examining the effects of hemodynamic forces, flat monolayer cultures have been used, which fail to account for the spatial organization and flow characteristics of real blood vessels.

In our model, we established the formation of a 3D tubular structure mimicking a blood vessel, connected with a peristaltic pump to provide continuous laminar flow at physiologically relevant shear stress levels. This enabled the creation of a dynamic microenvironment in which ECs underwent characteristic morphological remodeling. In particular, under shear stress levels of 0.2 dyn/cm² and 1.3 dyn/cm², cells acquired a cobblestone morphology (see Fig. 2) consistent with previous findings (Krüger‐Genge et al., 2019; Rojas‐González et al., 2023) and started to align in the direction of flow after 24 hr, as described in earlier studies (Chiu et al., 2003; Tkachenko et al., 2013). Under static conditions, HUVECs do not undergo significant morphological changes, whereas under flow, they elongate, and F‐actin filaments begin to align in a flow direction. This alignment increases progressively with higher WSS levels and demonstrates upstream Golgi polarization, aligning the Golgi apparatus with the direction of flow. Fluid shear stress induces phosphorylation of TIE2, Akt, Erk1/2, and eNOS in ECs, highlighting the key role of shear stress in the regulation of ECs (Ansarizadeh et al., 2025). EC alignment is affected not only by shear stress (Choi & Seo, 2019; Rojas‐González et al., 2023; Zhang et al., 2014), but also by the material from which the microfluidic channel is made (Chu et al., 2023) and the duration of cellular exposure to shear stress, where full reorientation of nuclei was observed only 10 days after surgical intervention (Aird, 2007). It is also important to note that shear stress affects the expression of adhesion molecules, namely, suppresses this response, which was not observed under static conditions in co‐culture with smooth muscle cells (SMCs) (Chiu et al., 2003). Under normal conditions, adhesion molecules (VCAM‐1, ICAM‐1, E‐selectin, P‐selectin) are not expressed on the surface of ECs. Their expression is induced by pro‐inflammatory factors such as TNF, IL‐1β, or bacterial components (LPS) (Huang et al., 2021; Moore et al., 2021; Ogrodzinski et al., 2023). However, some studies have found their expression in unstimulated ECs *in vitro* (Jubeli et al., 2012). In our study, a statistically significant increase in E‐selectin expression was observed in response to 10 ng/ml TNF after 18 hr of stimulation under laminar flow (see Fig. 3), indicating that ECs retained their ability for inflammatory activation within the established environment.

Quantitative analysis of the cell population before and after TNF treatment showed a dose‐dependent reduction in EC number. The greatest decrease was observed at a concentration of 10 ng/ml, indicating a cytotoxic or desquamative effect of TNF. Additionally, analysis of cell area (see Fig. 4) revealed a significant increase in the groups treated with 5 and 10 ng/ml TNF, consistent with the literature on morphological activation of ECs (Stroka et al., 2012; Wang et al., 2020). In our case, the elongated shape of the cells, particularly at 10 ng/ml TNF, was confirmed by a decrease in circularity coefficient to <0.5, while cells in the control group remained more rounded. These morphological changes were statistically significant and consistent with previous findings: under static conditions at different TNF concentrations for 24 hr, HUVEC in 2D (Stroka et al., 2012) and 3D (Wang et al., 2020) culture exhibited an elongated morphology with a reduced aspect ratio.

The proposed dynamic *in vitro* model represents a promising tool for investigating the molecular mechanisms underlying endothelial dysfunction. By replicating key physiological conditions, including laminar shear stress and controlled chemical stimulation, the model provides a microenvironment that closely mimics the *in vivo* vascular niche. This enables high‐resolution analysis of endothelial responses under both basal and inflammatory conditions, which is critical for understanding the pathogenesis of cardiovascular and systemic inflammatory diseases.

### Time Considerations

The described protocol requires at least 3 consecutive days to complete, although the duration may vary depending on experimental goals and downstream applications.

On day 1 (evening), FN is introduced into the device to facilitate extracellular matrix coating. This step is followed by an ON incubation to ensure uniform surface functionalization, which is critical for optimal cell adhesion.

On day 2, cells are seeded into the coated device under sterile conditions in two steps. Sufficient time should be allowed for cell attachment and initial spreading, as this directly influences the stability and reproducibility of subsequent flow experiments. Depending on the cell type, this step may require adjustments in seeding density and incubation period.

On day 3, the VOC is connected to a peristaltic pump to initiate controlled laminar flow. The perfusion stage may last from several hours to multiple days, depending on the experimental design and research question. During this period, cells are exposed to physiological or pathological flow conditions, enabling the study of hemodynamic effects, cell–cell interactions, and vascular remodeling.

In addition, immunofluorescence analysis should be considered, as the duration of this step varies depending on the specific incubation protocols for primary and secondary antibodies. This stage may further extend the experiment's overall timeline and should be integrated into the planning process.

In summary, while the core protocol spans three days, the flow phase and subsequent analyses, such as immunofluorescence staining, introduce variability that can extend the total duration of the experiment and should be carefully planned in relation to the intended outcomes.

### Author Contributions

**Svitlana M. Palii**: Formal analysis; investigation; methodology; writing—original draft. **Anastasiia Voytovych**: Investigation. **Nadiya Muzyka**: Resources; writing—original draft. **Nuria Chantada**: Investigation. **Pablo J. Sáez**: Conceptualization; funding acquisition; project administration; supervision. **Ezequiel Álvarez**: Conceptualization; data curation; funding acquisition; project administration; supervision. **Oksana Shevchuk**: Conceptualization; funding acquisition; project administration; supervision.

### Conflict of Interest

The authors declare no conflict of interest.

## Data Availability

The data, tools, and material (or their source) that support the protocol are available from the corresponding author upon reasonable request.
